# 

**Published:** 1921-03-26

**Authors:** 


### Recent Official Publications.

(To be obtained at H.M. Stationery Office, Imperial House, Kingsway, W.C. 2. The prices include postage.)

Medical Research Council—Special Report No. 52, on the Occurrence of Intestinal Protozoa in the Inhabitants of Britain. 2s. 2d.

Special Report No. 58. T.N.T. Poisoning. 3s. 2d

National Health Insurance—Interim Report by Government Actuary on the Valuation of Assets and Liabilities of Approved Societies at December 31, 1918, showing summarised results up to December 24, 1920. 2d.

Account of Securities held for the Reduction of National Debt, forming part of National Health Insurance Fund. 1½d.

Reports upon Openings in Industry Suitable for Disabled Sailors and Soldiers, No. III. 3d.

Army Medical Service—Regulations for the Amendments. 1½d.

Ministry of Health: Report of Departmental Committee on Control of Certain Therapeutic Substances (Cmd. 1156). 3d.

Unemployment Insurance Act, Amendment Bill. 2d.

### The Book Market.

The receipt of the following is acknowledged :—

J. B. Lippincott & Co.: "Essentials of Medicine." By C. Phillips Emerson, M.D. 12s. 6d. net.

H. Frowde and Hodder and Stoughton: "Graphic Methods in Heart Disease." Second edition. By John Hay, M.D., F.R.C.P. 12s. 6d. net.

Shaw & Sons: "Clinical Disorders of the Heart Beat." By T. Lewis, M.D., F.R.S., D.Sc., F.R.C.P., C.B.E. Fifth edition.

J. Bale Sons & Danielsson, Ltd.: "Manual for Health Visitors and Infant-Welfare Workers." Edited by Mrs. Enid Eve. 10s. 6d. net.

E. Arnold: "Medicine for Nurses." By J. Henderson, M.D., Ch.B., F.R.F.P.S.G. 8s. 6d. net.

J. Wright & Sons, Ltd., Bristol: "Blood Pictures; an Introduction to Clinical Hæmatology." By C. Price-Jones, M.B. Second edition. 6s. 6d. net.

Sweet & Maxwell, Ltd.: "Tyssen's Charitable Bequests, 1921." Second edition. 25s. net.

Humphrey Milford, Oxford University Press: "A Physician's Anthology of English and American Poetry." By C. A. Wood, M.D., and F. H. Garrison, M.D. 8s. 6d. net.

J. B. Lippincott Co.: "Lippincott's Quick Reference Book for Medicine and Surgery." By G. E. Rehberger, A.B., M.D. £3 3s. net.

Phillip Allan & Co.: "The Art of Attaining High Health." By F. Walker, M.A. 5s. net.

Longmans, Green & Co.: "Hygiene." By J. L. Notter and R. H. Firth. Ninth edition. 10s. 6d. net.

### Gifts and Bequests.

Braintree Cottage Hospital has received a cheque for £1,000 from Miss Ruth Courtauld, of Gosfield.

Mr. John Unwin, of Liverpool, has left £100 each to the Stanley Hospital and the Bootle Borough Hospital.

The Royal Hospital and Home for Incurables, at Putney, has received ten guineas as a contribution from the "33 Club."

The National Sunday League have given £100 War Loan stock to the Royal Hospital and Home for Incurables, Putney.

Colonel H. P. Kirkwood, late R.E., President of the Royal Mineral Water Hospital, Bath, left £500 to that institution.

Ex-Alderman J. A. Knight, a survivor of the Balaclava Charge, of Wednesbury, left £50 to the Wednesbury District Nursing Association.

Mr. William Beattie, of Newcastle-on-Tyne, has left £200 each to the Royal Infirmary and the Fleming Memorial Hospital for Sick Children.

Salop Infirmary will receive £200, the Oswestry Cottage Hospital £100, and Ellesmere Cottage Hospital £100 under the will of the late Mr. C. R. Moore, of Ellesmere, Salop.

The Freemasons' Hospital, Fulham Road, where the testator died, has been left £25 by Mr. F. W. Eastman, of Hammersmith, an actor and member of the original Grand Guignol Company.

Preston Royal Infirmary has received from a number of locally employed metal workers a large "magnet," for the removal of metal fragments in eye cases which are due to the presence of such foreign bodies.

Mrs. Mary Ann Heale, of Bristol, has left the following legacies to hospitals of that city: Royal Infirmary, £50; Hospital for Women and Children, and Orthopædic Hospital for Crippled Children, £30 each.

The local Welfare Committee of the United Services Fund has presented £250 to the Endowment Fund of the Felixstowe and Walton Cottage Hospital as the outcome of the action of the ex-Service men of the district who voted their grant to the hospital.

The Great Northern Hospital has recently received £22 from Miss L. McDonald (proceeds of entertainment at Camden House School), £10 10s. from National Union of Teachers, £1 1s. from Mrs. Dadge Stansfield (Johannesburg), and £1 from Mr. W. A. Bonner (Buenos Aires).

The Chelsea Hospital for Women has received towards the building of its greatly needed Nurses' Home £100 from the executors of the late Mr. F. L. Harvey. Also £25 from the trustees of the late Robert Williams Edwards Trust.

Mr. W. A. W. Darley, a governor of the Bank of Ireland, has left £500 to the Magdalen Asylum, Dublin, and, after the death of his wife, £1,000 to the Royal Hospital for Consumption, Newcastle, Wicklow.

Subject to a life interest, Mr. W. J. Hood-Rowan, of Blundellsands, Lancs, has left £200 each to the Cottage Hospital, Blundellsands, the Pendlebury Children's Hospital, and the Royal Infirmary, Oldham, and part of the residue of the estate to the Liverpool Infirmary for Children and the Leasowe Children's Hospital.

Under the will of Baroness Wantage, widow of the first and last Baron Wantage, V.C., K.C.B., the hospitals which benefit are Wantage Cottage Hospital, Charing Cross, and the Radcliffe Infirmary, Oxford, which will receive £1,000 each, and £500 each has been left to the Devonshire Hospital, Buxton, the Metropolitan Convalescent Institution, and the Royal General Bathing Hospital, Margate.

Of the residue of the estate of Mr. W. T. Teague, of Dartmouth, formerly of Oporto, the following receive varying shares: South Devon and East Cornwall Hospital, Plymouth, Dartmouth Cottage Hospital, King Edward's Hospital Fund, Guy's Hospital, the London Hospital, the Middlesex Hospital, Royal London Orthopædic Hospital, the Devon and Cornwall Homœopathic and Ear and Throat Hospitals, and the Plymouth Eye Infirmary.

### Matrons' and Sisters' Appointments.

WHITEHAVEN AND WEST CUMBERLAND INFIRMARY.—Miss Elsie Henderson Webb has been appointed night sister at this hospital, where she was trained. She has been night sister at Hartlepools Hospital, and done military and private nursing work.

SOUTHMEAD INFIRMARY, BRISTOL.—Miss Millie Dilke has been appointed home sister. She was trained at the Portsmouth Union Infirmary, Milton, where she was later staff nurse, ward sister, assistant theatre sister, and night superintendent.

SOUTH LONDON HOSPITAL FOR WOMEN, CLAPHAM COMMON.—Miss Dorothy Gibson has been appointed sister. She was trained at the Middlesex and Evelina Hospitals, and has served as sister, night superintendent, and home sister in Queen Alexandra's Imperial Military Nursing Service Reserve.

NORTH LONSDALE HOSPITAL, BARROW-IN-FURNESS.—Miss Alice Leveson has been appointed sister. She was trained at Huddersfield Royal Infirmary, and has been sister at Newark General Hospital, and engaged in military nursing.

ROYAL WEST SUSSEX HOSPITAL, CHICHESTER.—Miss Irene Abrahams has been appointed night sister. She was trained at the Royal Infirmary, Sheffield, and the Jessop Hospital for Women.

CARDIFF UNION HOSPITAL.—Miss Hannah Evans has been appointed ward sister. She was trained at St. Leonard's Hospital, Shoreditch, where she was staff nurse. She holds the certificate of the Central Midwives Board.

ST. LEONARD'S HOSPITAL, SHOREDITCH.—Miss Mary T. Morgan has been appointed sister of the children's ward, Miss Edith Kelly sister of a women's ward, and Miss Elizabeth Ramage Tait maternity sister. All three were trained and have been staff nurses at St. Leonard's Hospital, and hold the certificate of the Central Midwives Board.

INSTITUTION INFIRMARY, HOLGATE, LINTHORPE, MIDDLESBROUGH.—Miss Harriet Walker has been appointed ward sister. She was trained at Middlesbrough Union Infirmary.

QUEEN MARY'S HOSPITAL FOR CHILDREN, CARSHALTON.—Miss Eva S. Jordan has been appointed sister. She was trained at the Royal Albert Hospital, Devonport, and at Queen Charlotte's Hospital. She holds the certificate of the Central Midwives Board, and has been staff nurse in charge of ward and theatre under Q.A.I.M.N.S.R.

CROYDON BOROUGH SANATORIUM, NORTH CHEAM.—Miss Sarah Williams has been appointed matron. She was trained at the Bristol General Hospital, has been assistant matron of the Royal Victoria Infirmary, Newcastle-on-Tyne, night sister at Croydon General Hospital, ward sister and temporary housekeeper at Swansea General Hospital, and matron T.F.N.S. of the 2nd Eastern General Hospital, Brighton, and of the 21st General Hospital, Alexandria.

### Personalia.

THE resident and honorary staff, with the sisters and nurses of the Cumberland Infirmary, on the invitation of the Matron, Miss Cardwell, gave a handsome "send off" to Miss Agnes Agnew, who is resigning her post as sister after many years' service in order to be married. Handsome gifts, consisting of a silver breakfast dish, shopping bag, and silver vase, with onion, carrot, and Scotch kail, according to Scotch custom, for the "first broth," were presented. Sister Agnew distinguished herself in the rescue work at the time of the Gretna railway disaster, and was Mentioned in the *Gazette*. Some very cordial things were said of her work and influence in the infirmary by Dr. Lediard and Miss Cardwell.

### Addendum.

IN the answer last week to a correspondent—"Derry"—under the head of "Irish Nurses' Association," it should have been stated that the address of the Nurses' Club (College of Nursing, Ltd.) is 54 Fitzwilliam Square, N. Dublin.

March 26, 1921] [Price Twopence

### The Hospital

The Workers' Newspaper of Administrative Medicine and Institutional Life, Administration, National Insurance and Health.

**RATES OF SUBSCRIPTION** (post free, payable in advance).
Three months, 3/3; six months, 6/6; twelve months, 13/-. (Should the cost of printing and paper as wel as increased postage, necessitate alteration of these rates, the period paid for will be reduced proportionately on unexpired subscriptions.) THE HOSPITAL "Index" to Volume LXVIII., April to September 1920, is now ready, price 1/1 per copy, post free. Address The Manager, THE HOSPITAL, "The Hospital" Building, 28 29 Southampton Street, Strand, London, W.C. 2.

### Manager's Notices.

***All letters on Business matters must be addressed to the Manager, "The Hospital," 28 and 29 Southampton Street, London, W.C.***

**NOW READY, "The Hospital" Index to Vol. LXVIII., April to September 1920. Price 1s. 1d. per Copy post free.**

Rates for Subscription (payable in advance).

| | | | |
|---|---|---|---|
| Three Months (including Postage) | ... ... | 3s. | 3d. |
| Six Months do. | ... ... | 6s. | 6d. |
| Twelve Months do. | ... ... | 13s. | 0d. |

Subscriptions, which may commence at any time, are payable in advance. Cheques and Post-Office Orders should be crossed London County and Westminster Bank, Covent Garden Branch, and made payable to the Manager of The Hospital.

Should the cost of printing and paper, as well as increased postage, necessitate alteration of these rates, the period paid for will be reduced proportionately on unexpired subscriptions.

**It is especially requested that remittances be made by Cheque or Postal Order, and in halfpenny stamps only when the amount is under sixpence.**

To ensure insertion, all advertisements for The Hospital must reach the Manager not later than Wednesday at noon in each case.

*SUBSCRIPTION FORM.*

*Please send me* The Hospital *for* .................*months, commencing with your issue of* ..........................., *for which please find enclosed* ............................

*Name* ..............................................

*Address* ..............................................

..............................................

..............................................

*Date* ..............................................

*To* .............................................. *Newsagent.*

**THE HOSPITAL.**

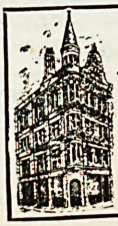

**The Modern Newspaper of Administrative Medicine and Institutional Life.**

**26th March, 1921.** **COUPON.**

